# Effects on Lung Gas Volume, Respiratory Mechanics and Gas Exchange of a Closed-Circuit Suctioning System during Volume- and Pressure-Controlled Ventilation in ARDS Patients

**DOI:** 10.3390/jcm10235657

**Published:** 2021-11-30

**Authors:** Davide Chiumello, Luca Bolgiaghi, Paolo Formenti, Tommaso Pozzi, Manuela Lucenteforte, Silvia Coppola

**Affiliations:** 1 Department of Anesthesia and Intensive Care, ASST Santi Paolo e Carlo, San Paolo University Hospital, Via di Rudini 9, 20122 Milan, Italy; luca.bolgiaghi@hotmail.it (L.B.); formenti.paolo80@gmail.com (P.F.); silvia_coppola@libero.it (S.C.); 2 Department of Health Sciences, University of Milan, 20122 Milan, Italy; tommaso.pozzi94@gmail.com (T.P.); manuela.lucenteforte@unimi.it (M.L.); 3 Coordinated Research Center on Respiratory Failure, University of Milan, 20122 Milan, Italy

**Keywords:** ARDS, endotracheal suctioning, EIT, closed-circuit suctioning

## Abstract

Mechanically ventilated patients periodically require endotracheal suctioning. There are conflicting data regarding the loss of lung gas volume caused by the application of a negative pressure by closed-circuit suctioning. The aim of this study was to evaluate the effects of suctioning performed by a closed-circuit system in ARDS patients during volume- or pressure-controlled ventilation. In this prospective crossover-design study, 18 ARDS patients were ventilated under volume and pressure control applied in random order. Gas exchange, respiratory mechanics and EIT-derived end-expiratory lung volume (EELV) before the suctioning manoeuvre and after 5, 15 and 30 min were recorded. The tidal volume and respiratory rate were similar in both ventilation modes; in volume control, the EELV decreased by 31 ± 23 mL, 5 min after the suctioning, but it remained similar after 15 and 30 min; the oxygenation, PaCO_2_ and respiratory system elastance did not change. In the pressure control, 5 min after suctioning, EELV decreased by 35 (26–46) mL, the PaO_2_/FiO_2_ did not change, while PaCO_2_ increased by 5 and 30 min after suctioning (45 (40–51) vs. 48 (43–52) and 47 (42–54) mmHg, respectively). Our results suggest minimal clinical advantages when a closed system is used in volume-controlled compared to pressure-controlled ventilation.

## 1. Introduction

The ventilatory management of ARDS patients should ensure adequate PEEP levels and lung recruitment in order to prevent alveolar collapse and the development of VILI [1,2]. Generally, mechanically ventilated patients require periodical endotracheal suctioning to prevent the accumulation of secretions in the respiratory system [3]. However, endotracheal suctioning is an invasive procedure possibly associated with several side effects such as the loss of mean airway pressure, atelectasis, oxygen desaturation, tissue trauma, arrythmias, ventilator asynchronies and bronchospasms [4]. Leur et al. reported complications associated with routine endotracheal suctioning in up to 39% of mechanically ventilated patients [5]. Similarly, in a one-year-long large observational study, Maggiore et al. found a rate of complications in 40% of patients, with oxygen desaturation and haemorrhagic secretions reported in 35% and 12% of cases, respectively. Furthermore, they identified a PEEP level higher than 5 cmH_2_O and the diagnosis of ARDS as independently associated risk factors for oxygen desaturation [6]. By applying the quantitative lung computed tomography scan analysis in a group of sedated and paralyzed patients with acute respiratory failure, it was reported that patient disconnection and suctioning were associated with a reduction in lung gas volume of up to 27%, leading to atelectasis. The reduction in lung volume was also associated with a decrease in arterial oxygenation [6].

In order to limit these possible side effects, closed-circuit suctioning systems have been suggested [3]. A closed-circuit suctioning system is composed of a catheter which is directly introduced into the airway without any disconnection, allowing the continuous delivery of tidal volume and thus the maintenance of minute ventilation and mean airway pressure [3,7]. However, applying a negative pressure into the closed-circuit system generates a flow which could exceed the minute ventilation with possible autocycling/desynchrony with the ventilator, promoting lung derecruitment [8]. Currently, there are conflicting data regarding the possible loss of lung gas volume due to closed-circuit suctioning. Some studies have shown a loss of lung gas volume related to the procedure that was almost recovered after 10 min [6,9], while others have reported that this volume loss was detectable even after 20–30 min [10,11]. However, the study population, the suctioning technique, the length of suctioning, the type of catheter and the lung volume measurement method significantly differed among the studies.

In order to minimize the risk of gas volume loss, recruitment manoeuvres and different ventilator settings have been suggested [12,13]. Two experimental animal studies showed that the use of volume-controlled compared to pressure-controlled ventilation resulted in higher arterial oxygenation and lung volume [14,15].

The aim of this study was to evaluate the effects of suctioning performed by a closed-circuit system in terms of lung gas volume, respiratory mechanics and gas exchange in ARDS patients during volume- and pressure-controlled ventilation.

## 2. Materials and Methods

### 2.1. Study Population

This prospective crossover-design study was conducted from June 2018 to August 2021 in the intensive care unit (ICU) of San Paolo University Hospital (ASST Santi Paolo Carlo, Milan, Italy). The protocol was approved by the local ethical committee and informed consent was obtained according to Italian regulations. Orotracheally intubated and mechanically ventilated patients with a diagnosis of ARDS according to the Berlin definition were enrolled [16]. Exclusion criteria were: age less than 18 years, barotrauma, hemodynamic instability, the presence of cardiac pacing and/or a thoracic drainage, and history of severe chronic obstructive pulmonary disease.

### 2.2. Study Protocol

**Figure 1:**
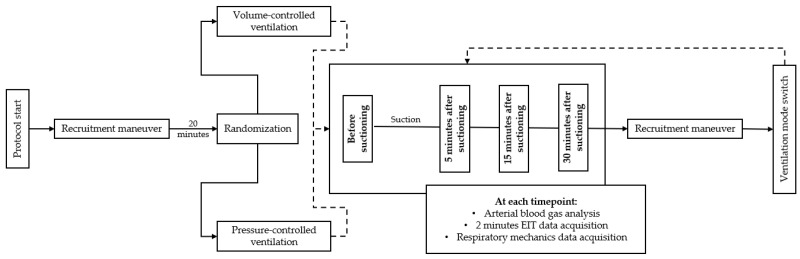
Study protocol. EIT: electrical impedance tomography.

The flow chart of this study is shown in Figure 1. All patients were in supine position, deeply sedated, paralyzed and ventilated under volume control with a tidal volume of 6–8 mL/kg of ideal body weight (IBW). The respiratory rate was selected to maintain a PaCO_2_ between 40 and 50 mmHg; the oxygen inspired fraction (FiO_2_) and the PEEP level were selected to maintain a peripheral oxygen saturation (SpO_2_) >88%. A recruitment manoeuvre was applied in pressure-controlled ventilation with a PEEP of 5 cmH_2_O at an inspiratory plateau pressure of 45 cmH_2_O with a respiratory rate of 10 for two minutes to standardize the lung volume [17]. After 20 min, volume-controlled (VCV) or pressure-controlled ventilation (PCV) was randomly selected and each patient underwent one of the two modes of ventilation for 30 min.

The endotracheal suctioning was performed after 5 min from the start of the randomized ventilation mode by applying a closed-circuit system (Closed Suction System for adults, Halyard, Alpharetta, GA, USA). The suctioning catheter was inserted into the endotracheal tube and advanced until resistance was met; it was then withdrawn by 2–3 cm. A negative pressure of 150 cmH_2_O was continuously applied for 8 s, the catheter was then withdrawn and the suctioning was withheld for 6 s; subsequently, another suctioning procedure of 8 s was performed. No hyperoxygenation or recruitment manoeuvres were applied after the suctioning.

During the 30 min of ventilation, arterial blood gas analysis, measurements of changes in the end-expiratory lung gas volume and of respiratory mechanics were performed.

At the end of the 30 min of the first ventilation mode, a recruitment manoeuvre was applied and after a washout interval of 20 min, the other mode of ventilation was applied with the same protocol. The applied PEEP, tidal volume and oxygen fraction were similar between the ventilation modes.

### 2.3. Data Analysis

Before the endotracheal suctioning (after 3 min from the start of the randomized ventilation mode) and after 5, 15 30 min following the procedure, an arterial blood gas analysis was performed. In the last two minutes of each measurement period, changes in the end-expiratory lung volume (EELV) were measured by changes in the end-expiratory lung impedance using the Electric Impedance Tomography technique (PulmoVista, Dräger Italia, Milan, Italy). The principle of EIT imaging was already described in detail [18]: briefly, the EIT output image was based on the estimation of the impedance changes occurring inside the lung during inflation. Sixteen electrodes were placed around the thorax at the fifth–sixth intercostal places and connected to the EIT monitor. In the last 1 min, two inspiratory and expiratory pauses were performed to measure the static airway pressure. Respiratory mechanics (driving pressure, respiratory system elastance) was computed accordingly to the standard equation under static conditions [19].

### 2.4. Statistical Analysis

We calculated that a sample of 18 patients would constitute the trial with 80% power to show a reduction of 10% in the end-expiratory lung volume after the suctioning procedure at a two-sided α level of 0.05 [15]. Categorical data are reported as % (number), while continuous variables are expressed as the mean ± SD or median (IQR), as appropriate; normality of distribution was assessed by the Shapiro–Wilks tests. A one-way analysis of variance (ANOVA) for repeated measures or the Friedman test were used to assess differences within timepoints in each ventilation mode as appropriate; if a difference was found, a Tukey test was used for post hoc comparisons. A two-way ANOVA for repeated measures or a linear mixed effect model were used to analyse the effect of time and ventilation mode on the selected variables as appropriate. All analysis were performed with R Studio (R Foundation for Statistical Computing, Vienna, Austria).

## 3. Results

Eighteen patients were enrolled. The baseline clinical characteristics of the population are reported in Table 1. The mean age and body mass index were 68 ± 16 years and 25 ± 4 kg/m^2^, respectively. All patients were endotracheally intubated with a tube of an internal diameter of 7.5 (7.5–8.0) mm. The median time from admission to the day of the study was 2 (2–3) days. At baseline, the elastance of the respiratory system was 21 (18–26) cmH_2_O/L, PaO_2_/FiO_2_ and PEEP were 161 ± 36 and 10 ± 3 cm H_2_O, respectively.

### 3.1. Voume-Controlled Ventilation

**Figure 2:**
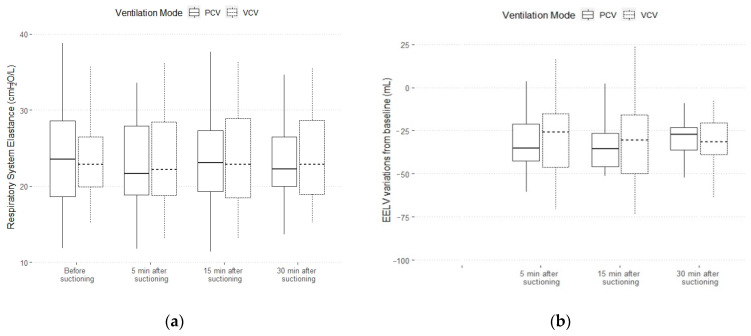
(**a**) Respiratory system elastance variations within timepoints in volume- and pressure-controlled ventilation; (**b**) end-expiratory lung volume (EELV) difference from “before suctioning” timepoint variations within timepoints in volume- and pressure-controlled ventilation. A Friedman test was used to test the differences within timepoints. No statistical difference within timepoints for each variable was found.

In VCV, the mean applied tidal volume 489 ± 60 mL and the respiratory rate of 17 ± 3 bpm remained unchanged throughout this study (Table 2). Before suctioning, the PaO_2_/FiO_2_ and PaCO_2_ were 168 (132–184) and 47 ± 9 mmHg. Five minutes after suctioning, the EELV decreased by 31 ± 23 mL, whilst the PaO_2_/FiO_2_ and the PaCO_2_ did not change. After 15 and 30 min, the EELV variation from the “before suctioning timepoint”, the respiratory system elastance and the PaCO_2_ remained similar (Figure 2).

### 3.2. Pressure-Controlled Ventilation

In PCV, the applied tidal volume and respiratory rate were similar within the timepoint (Table 3). Before suctioning, the PaO_2_/FiO_2_ and PaCO_2_ were 174 ± 38 and 45 (40–51) mmHg. Five minutes after the suctioning, the EELV decreased by 35 (26–46) mL, the PaO_2_/FiO_2_ did not change, while the PaCO_2_ significantly increased (45 (40–51) vs. 48 (43–52) mmHg). After 30 min, the PaCO_2_ was significantly higher (45 (40–51) vs. 47 (42–54) mmHg), while the EELV variation respiratory system elastance did not change (Figure 2).

## 4. Discussion

The main findings of this study evaluating the effects of closed-circuit suctioning during volume-controlled compared to pressure-controlled ventilation in a group of ARDS patients were: (1) the end-expiratory lung volume did not change after suctioning in both ventilation modes; and (2) oxygenation was not clinically affected while the arterial carbon dioxide slightly increased during the study under pressure-controlled ventilation.

Endotracheal suctioning is one of the most frequently applied procedures in order to prevent airway obstruction and enhance secretion clearance from the respiratory tract in mechanically ventilated patients. However, the suctioning can cause lung collapse, atelectasis, desaturation and hemodynamic disturbances by generating a negative pressure inside the respiratory system [3].

The two commonly applied methods are those of open and closed-circuit suctioning; while the first requires the disconnection of the patient from the ventilator to allow the insertion of the suctioning catheter, in the latter, the catheter is inside the ventilator circuit, thus avoiding any disconnection during the procedure. The theoretically possible advantages of closed-circuit suctioning include a lower decrease in oxygen saturation, a better maintenance of PEEP and a smaller loss of lung volume [7]. Cereda et al. showed that during suctioning in a population of ARDS patients ventilated with a mean PEEP level of 10 cmH_2_O, the decrease in lung gas volume was significantly lower when using closed compared to open-circuit suctioning [20]. Similarly, Maggiore et al. found a significantly higher reduction in lung gas volume (1.4 L vs. 0.5 L) in a group of ARDS patients: the higher the applied external PEEP was, the higher the loss of gas volume. After one minute of suctioning, the loss of gas volume was almost recovered only when closed-circuit suctioning was used [21]. A recent systematic review found a significantly higher decrease in oxygen saturation immediately after suctioning with the open technique, while no changes or an increase occurred with the closed technique [7].

Thus, closed-circuit suctioning should be considered for treating patients at high risk of desaturation under conditions of high PEEP levels and a high inspired oxygen fraction [3]. Furthermore, in order to limit the possible negative effects of closed-circuit suctioning, the applied negative suctioning pressure should not exceed 150 mmHg with a duration less than 15 s. Accordingly, in the present study, we used closed-circuit suctioning with a negative pressure of 150 cmH2O and a total procedure duration of less than 10 s.

However, the potential risk of closed-circuit suctioning consists of the mechanical ventilator not being able to replace the amount of gas aspirated during suctioning. In an experimental setting with closed-circuit suctioning, volume-controlled ventilation caused a significantly higher generation of intrinsic PEEP compared to pressure-controlled ventilation while the minute ventilation was similarly reduced [22]. Corley et al. found that a closed-circuit, despite minimizing the loss of gas volume during suctioning, resulted in a slower recovery of lung volume after the manoeuvre in 20 post-cardiac surgery patients [11].

To limit these negative effects, a recruitment manoeuvre after suctioning or different ventilatory settings have been suggested [9,11,12,13].

Few data are exist regarding the effects of a closed-circuit comparing volume- and pressure-controlled ventilation on respiratory mechanics and gas exchange. In the present study, we used EIT, which is a non-invasive, radiation-free technique which allows one to monitor the changes and the distribution of gas into the lung in real time. A strong correlation was reported between the changes in the end-expiratory lung gas volume and the expiratory lung impedance computed by the EIT [18].

We did not measure the changes in lung gas volume during the suctioning procedure, but 5, 15 and 30 min after mechanical ventilation was resumed. In both ventilation modes, we did not observe any changes in the end-expiratory lung gas volume. On the contrary, Heinz et al. found that, in post-operative patients, both in pressure- and volume-controlled ventilation, the end-expiratory lung gas volume was slightly lower 20 min after the suctioning manoeuvre (3.3 vs. 3.2 L and 3.3 vs. 3.2 L) [10]. The duration of suctioning, as well as the negative applied pressure and the diameter of the catheter, were maintained to be the lowest possible according to previous suggestions [3].

Concerning the gas exchange, we did not find any clinically relevant difference in arterial oxygenation in both ventilation modes while the arterial carbon dioxide increased significantly during pressure-controlled ventilation. Previous animal and human studies showed a post-suctioning increase in carbon dioxide after 10 and 30 min [14,23]. In the present study, the increase in arterial carbon dioxide could be due to a decrease in alveolar ventilation (i.e., an increase in dead space), this being the minute ventilation and hemodynamic which are similar in pressure and volume-controlled ventilation.

The possible limitations of this study are: (1) the absence of any data regarding the quantity of removed secretions; and (2) the impossibility of transferring these data during assisted ventilation because all the patients were sedated and paralyzed.

## 5. Conclusions

These results suggest minimal clinical advantages when closed-circuit suctioning is used in volume-controlled compared to pressure-controlled ventilation in terms of possible end-expiratory lung gas volume and gas exchange variations. Thus, when a closed-circuit is used, it does not require any changes in the ventilatory settings or recruitment manoeuvres to prevent possible negative effects.

## Figures and Tables

**Table 1:** Baseline characteristics of the study population.

Characteristics	*n* = 18
Age, *years*	68 ± 16
Male sex, *% (n)*	61 (11)
Weight, *kg*	73 ± 13
Height, *cm*	170 (163–178)
BMI, *kg/m*^2^	25 ± 4
Pulmonary ARDS, *% (n)*	71 (10)
Extraulmonary ARDS, *% (n)*	29 (8)
Mild–moderate ARDS, *% (n)*	62 (11)
Moderate–severe ARDS, *% (n)*	38 (7)
Time from ICU admission to study day, *days*	2 (2–3)
Tube diameter, *mm*	7.5 (7.5–8.0)
Tidal volume, *mL*	477 ± 64
Tidal volume per ideal body weight, *mL/kg*	7.2 ± 0.4
Respiratory rate, *bpm*	17 ± 3
Minute ventilation, *L/min*	8.0 ± 2.0
Peak pressure, *cmH*_2_*O*	30 ± 6
Plateau pressure, *cmH*_2_*O*	21 ± 4
PEEP, *cmH*_2_*O*	10 ± 3
Driving pressure, *cmH*_2_*O*	11 (8–13)
Respiratory system elastance, *cmH*_2_*O/L*	21 (18–26)
PaCO_2_, *mmHg*	45 (41–47)
PaO_2_, *mmHg*	77 ± 8
PaO_2_/FiO_2_	161 ± 36

Quantitative data are presented as the mean ± SD or median (IQR) as appropriate; the categorical variables are expressed as % (n). BMI: body mass index; PEEP: positive end-expiratory pressure; PaCO_2_: carbon dioxide arterial partial pressure; PaO_2_: oxygen arterial partial pressure; PaO_2_/FiO_2_: oxygen arterial partial pressure on inspired fraction of oxygen ratio.

**Table 2:** Respiratory mechanics, gas exchange and electrical impedance tomography data within timepoints when patients were ventilated in volume-controlled mode.

Characteristics	Before Suctioning	5 Min after Suctioning	15 Min after Suctioning	30 Min after Suctioning	*p*-Value
Tidal volume, *mL*	489 ± 60	489 ± 60	489 ± 60	489 ± 60	*-------*
Respiratory rate, *bpm*	17 ± 3	17 ± 3	17 ± 3	17 ± 3	*-------*
Minute ventilation, *L/min*	8.2 ± 1.7	8.2 ± 1.7	8.2 ± 1.7	8.2 ± 1.7	*-------*
Peak pressure, *cmH*_2_*O*	29 ± 5	30 ± 5	29 ± 6	29 ± 5	0.505
Plateau pressure, *cmH*_2_*O*	22 ± 4	22 ± 4	22 ± 4	22 ± 4	0.699
Driving pressure, *cmH*_2_*O*	11 (8–13)	11 (8–13)	11 (8–12)	11 (8–13)	0.607
Mean airway pressure, *cmH*_2_*O*	15 (14–16)	14 (14–17)	14 (14–16)	14 (14–16)	0.758
Respiratory system elastance, *cmH*_2_*O/L*	22 (17–26)	22 (16–27)	21 (16–27)	22 (17–27)	0.531
Mechanical power, *J/min*	15.5 ± 5.4	15.9 ± 5.3	15.6 ± 5.4	15.9 ± 5.2	0.403
PaCO_2_, *mmHg*	47 ± 9	49 ± 10	49 ± 10	50 ± 11	0.091
Ventilatory Ratio	1.4 (1.2–2.0)	1.6 (1.3–2.0)	1.6 (1.3–2.0)	1.6 (1.3–1.9)	0.085
PaO_2_, *mmHg*	77 (71–84)	78 (73–91)	80 (74–93) *	77 (75–88)	**0.020**
PaO_2_/ FiO_2_, *mmHg*	168 (132–184)	170 (134–189)	177 (140–196) *	178 (134–198)	**0.011**
EELV variations from baseline, *mL*		−31 ± 23	−32 ± 16	−31 ± 22	0.876

*: *p* < 0.05 vs. before suction timepoint; *p* < 0.05 vs. 5 min after suction timepoint; *p* < 0.05 vs. 15 min after suction timepoint.

**Table 3:** Respiratory mechanics, gas exchange and electrical impedance tomography data within timepoints when patients were ventilated in pressure-controlled mode.

Characteristics	Before Suctioning	5 Min after Suctioning	15 Min after Suctioning	30 Min after Suctioning	*p*-Value
Tidal volume, *mL*	480 ± 60	480 ± 65	485 ± 56	485 ± 51	0.680
Respiratory rate, *bpm*	17 ± 3	17 ± 3	17 ± 3	17 ± 3	-------
Minute ventilation, *L/min*	8.0 ± 1.7	8.0 ± 1.5	8.1 ± 1.6	8.1 ± 1.6	0.928
Δ Pressure over PEEP, *cmH_2_O*	17 ± 4	17 ± 4	17 ± 4	17 ± 4	-------
Mean airway pressure, *cmH_2_O*	15 ± 3	15 ± 3	15 ± 3	15 ± 3	0.820
Respiratory system elastance, *cmH_2_O/L*	24 ± 7	24 ± 8	24 ± 8	23 ± 7	0.493
Mechanical power, *J/min*	22.0 ± 5.6	21.8 ± 5.7	22.4 ± 5.7	22.5 ± 5.8	0.408
PaCO_2_, *mmHg*	45 (40–51]	48 (43–52) *	47 (42–50)	47 (42–54) *	**0.004**
Ventilatory Ratio	1.4 (1.2–2.0]	1.6 (1.3–2.0)	1.6 (1.3–2.0)	1.6 (1.3–1.9)	**0.078**
PaO_2_, *mmHg*	84 (76–93]	79 (74–9)	80 (75–93)	79 (76–91)	0.419
PaO_2_/ FiO_2_, *mmHg*	174 ± 38	172 ± 43	171 ± 36	171 ± 36	0.410
EELV variations from baseline, *mL*		−35 (−46–−26)	−27 (−36–−23)	−35 (−43–−21)	0.908

*: *p* < 0.05 vs. before suction timepoint; *p* < 0.05 vs. 5 min after suction timepoint; *p* < 0.05 vs. 15 min after suction timepoint.
